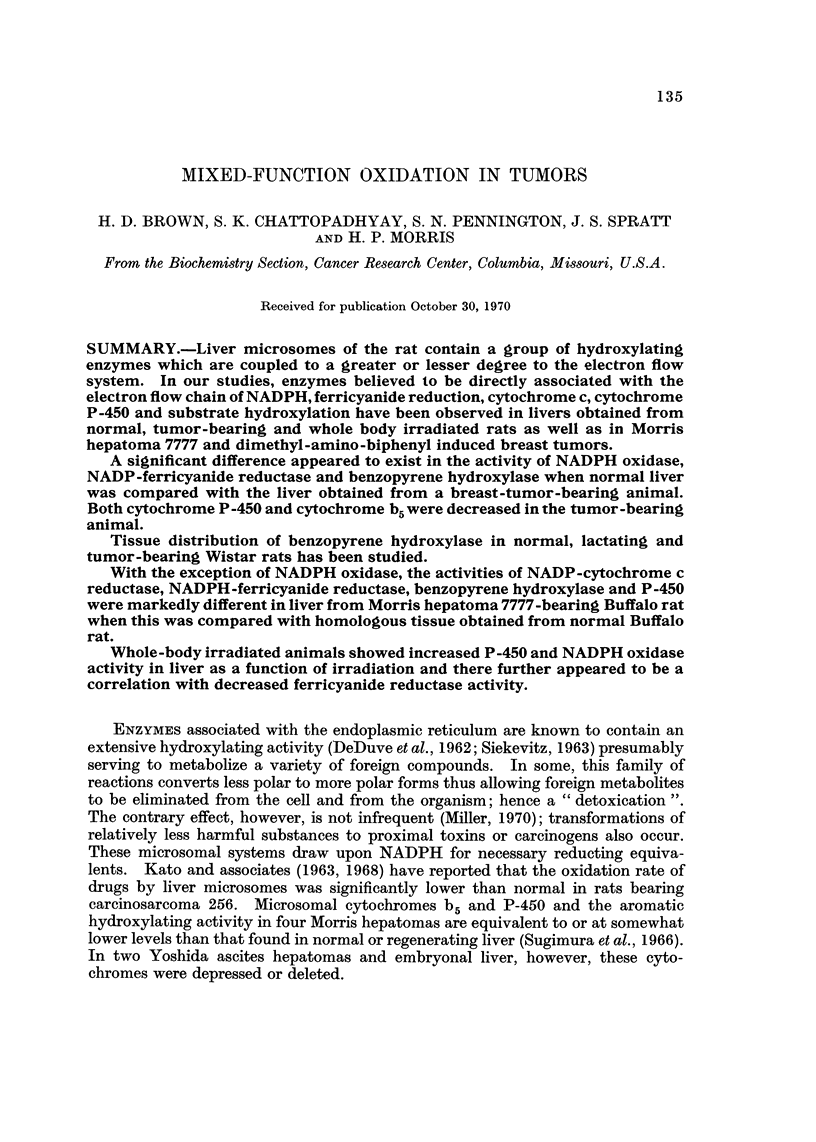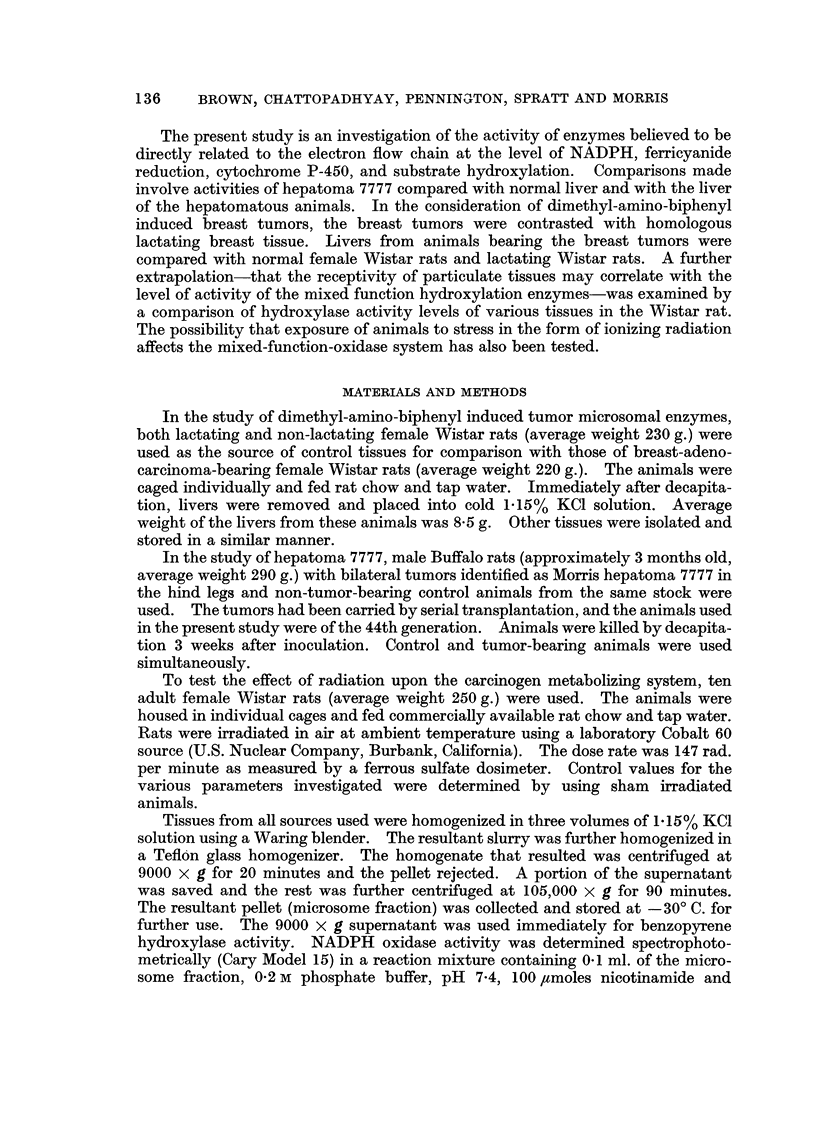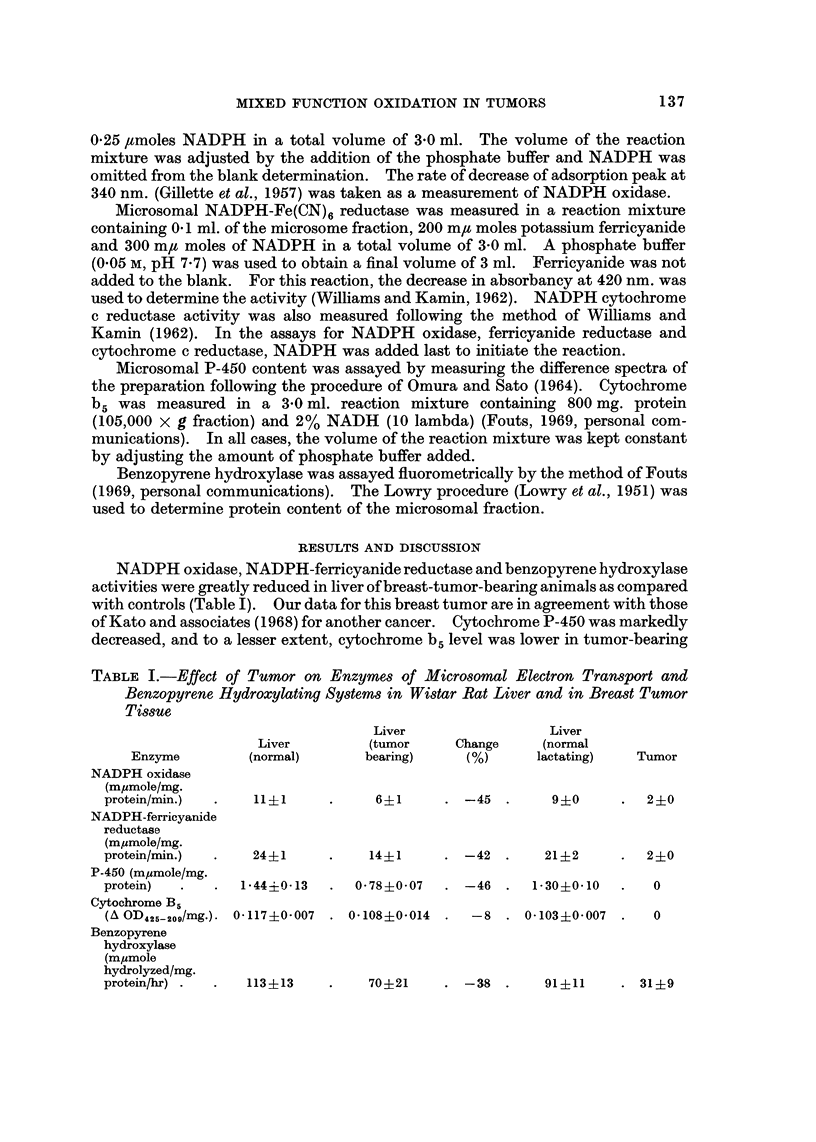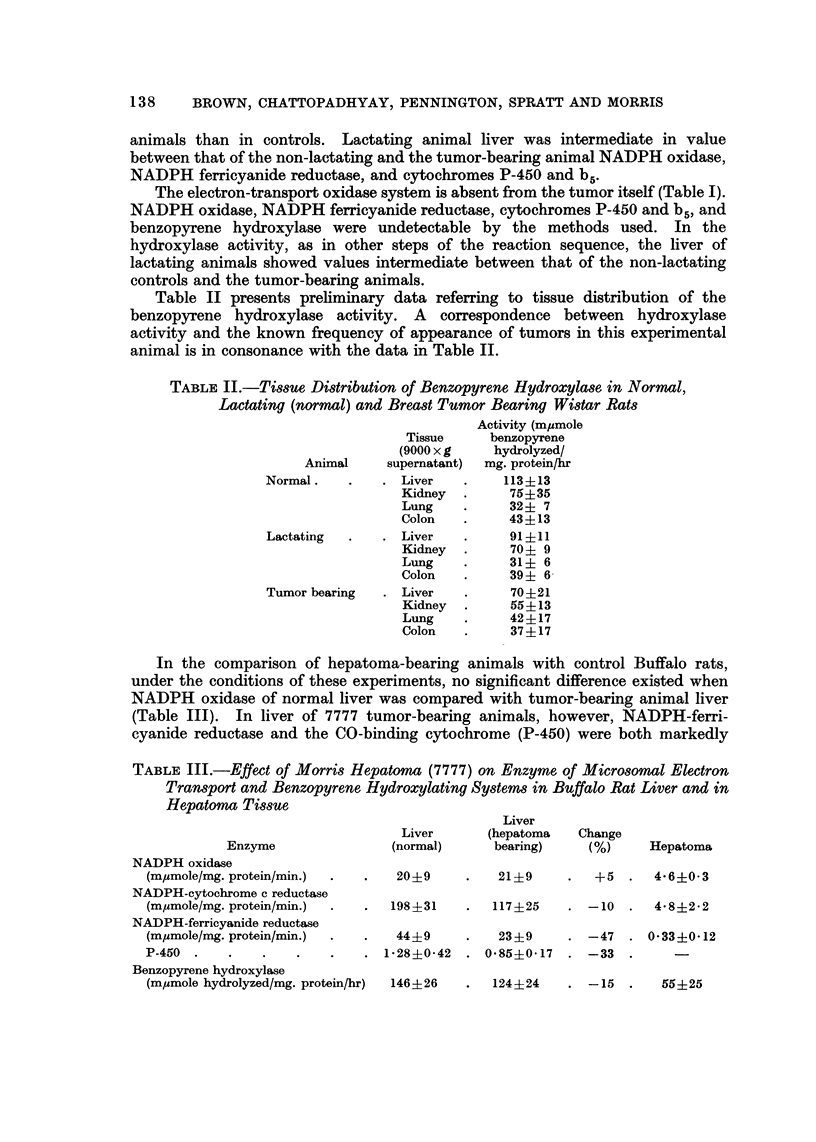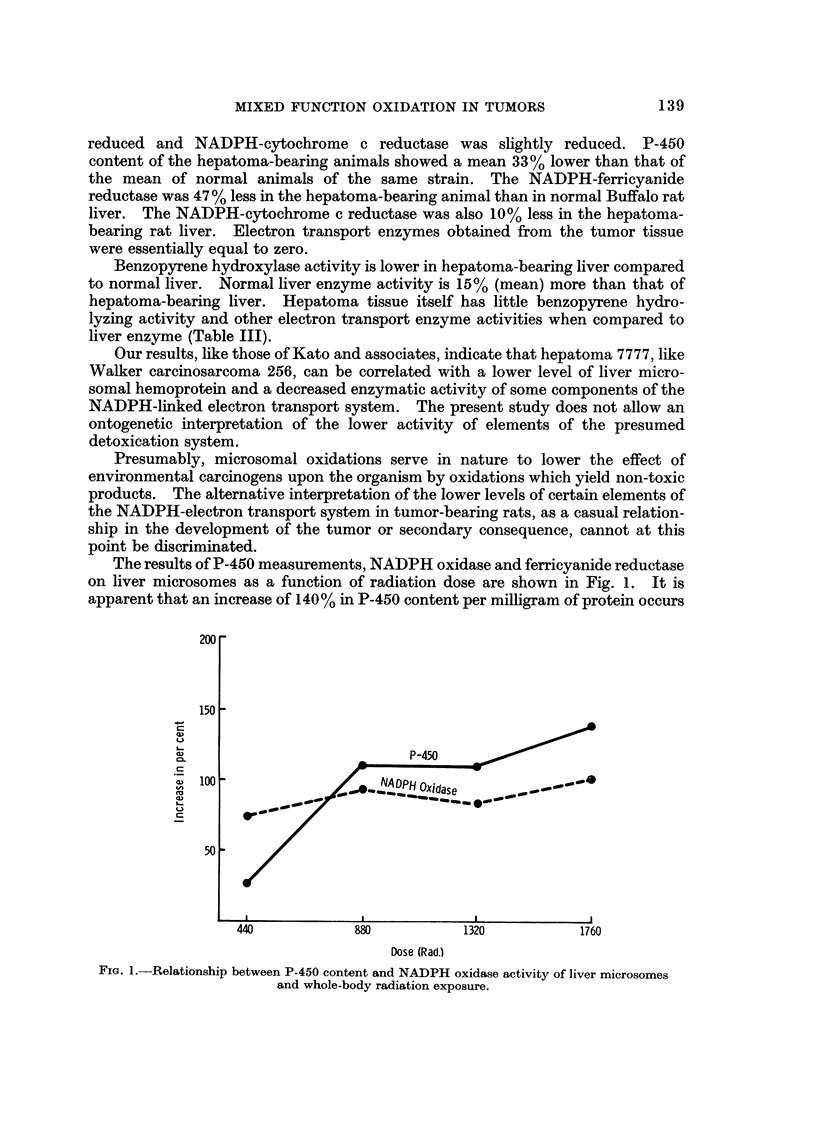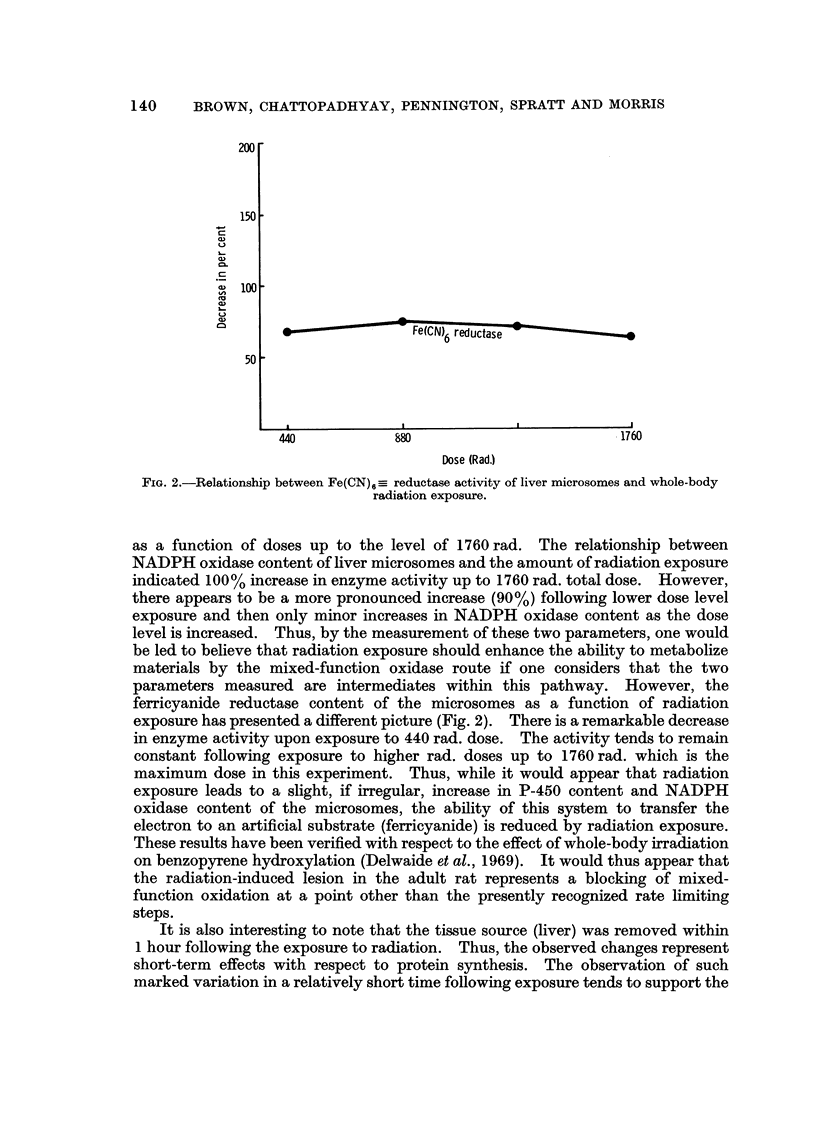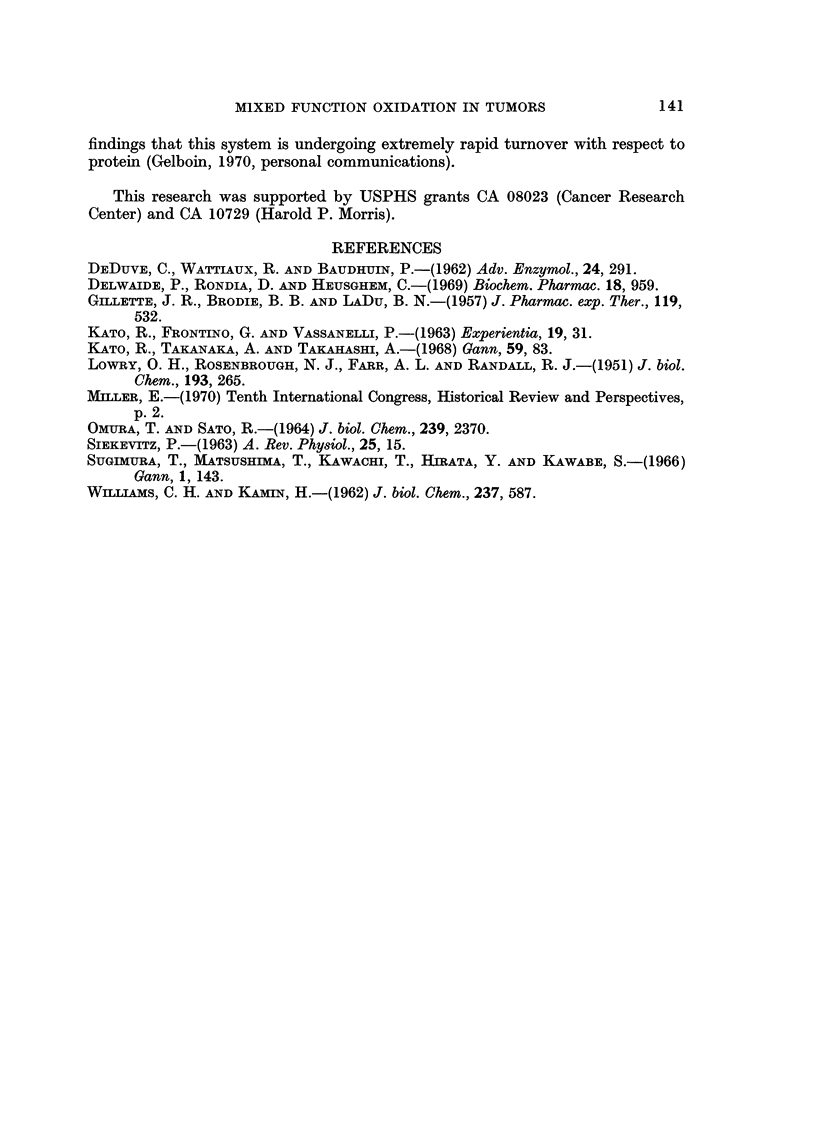# Mixed-Function Oxidation in Tumors

**DOI:** 10.1038/bjc.1971.19

**Published:** 1971-03

**Authors:** H. D. Brown, S. K. Chattopadhyay, S. N. Pennington, J. S. Spratt, H. P. Morris

## Abstract

Liver microsomes of the rat contain a group of hydroxylating enzymes which are coupled to a greater or lesser degree to the electron flow system. In our studies, enzymes believed to be directly associated with the electron flow chain of NADPH, ferricyanide reduction, cytochrome c, cytochrome P-450 and substrate hydroxylation have been observed in livers obtained from normal, tumor-bearing and whole body irradiated rats as well as in Morris hepatoma 7777 and dimethyl-amino-biphenyl induced breast tumors.

A significant difference appeared to exist in the activity of NADPH oxidase, NADP-ferricyanide reductase and benzopyrene hydroxylase when normal liver was compared with the liver obtained from a breast-tumor-bearing animal. Both cytochrome P-450 and cytochrome b_5_ were decreased in the tumor-bearing animal.

Tissue distribution of benzopyrene hydroxylase in normal, lactating and tumor-bearing Wistar rats has been studied.

With the exception of NADPH oxidase, the activities of NADP-cytochrome c reductase, NADPH-ferricyanide reductase, benzopyrene hydroxylase and P-450 were markedly different in liver from Morris hepatoma 7777-bearing Buffalo rat when this was compared with homologous tissue obtained from normal Buffalo rat.

Whole-body irradiated animals showed increased P-450 and NADPH oxidase activity in liver as a function of irradiation and there further appeared to be a correlation with decreased ferricyanide reductase activity.


### MIXED-FUNCTION OXIDATION IN TUMORS

H. D. BROWN, S. K. CHATTOPADHYAY, S. N. PENNINGTON, J. S. SPRATT AND H. P. MORRIS

*From the Biochemistry Section, Cancer Research Center, Columbia, Missouri, U.S.A.*

Received for publication October 30, 1970

**SUMMARY.—Liver microsomes of the rat contain a group of hydroxylating enzymes which are coupled to a greater or lesser degree to the electron flow system. In our studies, enzymes believed to be directly associated with the electron flow chain of NADPH, ferricyanide reduction, cytochrome c, cytochrome P-450 and substrate hydroxylation have been observed in livers obtained from normal, tumor-bearing and whole body irradiated rats as well as in Morris hepatoma 7777 and dimethyl-amino-biphenyl induced breast tumors.**

**A significant difference appeared to exist in the activity of NADPH oxidase, NADP-ferricyanide reductase and benzopyrene hydroxylase when normal liver was compared with the liver obtained from a breast-tumor-bearing animal. Both cytochrome P-450 and cytochrome $b_5$ were decreased in the tumor-bearing animal.**

**Tissue distribution of benzopyrene hydroxylase in normal, lactating and tumor-bearing Wistar rats has been studied.**

**With the exception of NADPH oxidase, the activities of NADP-cytochrome c reductase, NADPH-ferricyanide reductase, benzopyrene hydroxylase and P-450 were markedly different in liver from Morris hepatoma 7777-bearing Buffalo rat when this was compared with homologous tissue obtained from normal Buffalo rat.**

**Whole-body irradiated animals showed increased P-450 and NADPH oxidase activity in liver as a function of irradiation and there further appeared to be a correlation with decreased ferricyanide reductase activity.**

ENZYMES associated with the endoplasmic reticulum are known to contain an extensive hydroxylating activity (DeDuve *et al.*, 1962; Siekevitz, 1963) presumably serving to metabolize a variety of foreign compounds. In some, this family of reactions converts less polar to more polar forms thus allowing foreign metabolites to be eliminated from the cell and from the organism; hence a " detoxication ". The contrary effect, however, is not infrequent (Miller, 1970); transformations of relatively less harmful substances to proximal toxins or carcinogens also occur. These microsomal systems draw upon NADPH for necessary reducting equivalents. Kato and associates (1963, 1968) have reported that the oxidation rate of drugs by liver microsomes was significantly lower than normal in rats bearing carcinosarcoma 256. Microsomal cytochromes $b_5$ and P-450 and the aromatic hydroxylating activity in four Morris hepatomas are equivalent to or at somewhat lower levels than that found in normal or regenerating liver (Sugimura *et al.*, 1966). In two Yoshida ascites hepatomas and embryonal liver, however, these cytochromes were depressed or deleted.

The present study is an investigation of the activity of enzymes believed to be directly related to the electron flow chain at the level of NADPH, ferricyanide reduction, cytochrome P-450, and substrate hydroxylation. Comparisons made involve activities of hepatoma 7777 compared with normal liver and with the liver of the hepatomatous animals. In the consideration of dimethyl-amino-biphenyl induced breast tumors, the breast tumors were contrasted with homologous lactating breast tissue. Livers from animals bearing the breast tumors were compared with normal female Wistar rats and lactating Wistar rats. A further extrapolation—that the receptivity of particulate tissues may correlate with the level of activity of the mixed function hydroxylation enzymes—was examined by a comparison of hydroxylase activity levels of various tissues in the Wistar rat. The possibility that exposure of animals to stress in the form of ionizing radiation affects the mixed-function-oxidase system has also been tested.

### MATERIALS AND METHODS

In the study of dimethyl-amino-biphenyl induced tumor microsomal enzymes, both lactating and non-lactating female Wistar rats (average weight 230 g.) were used as the source of control tissues for comparison with those of breast-adeno-carcinoma-bearing female Wistar rats (average weight 220 g.). The animals were caged individually and fed rat chow and tap water. Immediately after decapitation, livers were removed and placed into cold 1·15% KCl solution. Average weight of the livers from these animals was 8·5 g. Other tissues were isolated and stored in a similar manner.

In the study of hepatoma 7777, male Buffalo rats (approximately 3 months old, average weight 290 g.) with bilateral tumors identified as Morris hepatoma 7777 in the hind legs and non-tumor-bearing control animals from the same stock were used. The tumors had been carried by serial transplantation, and the animals used in the present study were of the 44th generation. Animals were killed by decapitation 3 weeks after inoculation. Control and tumor-bearing animals were used simultaneously.

To test the effect of radiation upon the carcinogen metabolizing system, ten adult female Wistar rats (average weight 250 g.) were used. The animals were housed in individual cages and fed commercially available rat chow and tap water. Rats were irradiated in air at ambient temperature using a laboratory Cobalt 60 source (U.S. Nuclear Company, Burbank, California). The dose rate was 147 rad. per minute as measured by a ferrous sulfate dosimeter. Control values for the various parameters investigated were determined by using sham irradiated animals.

Tissues from all sources used were homogenized in three volumes of 1·15% KCl solution using a Waring blender. The resultant slurry was further homogenized in a Teflon glass homogenizer. The homogenate that resulted was centrifuged at 9000 $\times$ $g$ for 20 minutes and the pellet rejected. A portion of the supernatant was saved and the rest was further centrifuged at 105,000 $\times$ $g$ for 90 minutes. The resultant pellet (microsome fraction) was collected and stored at $-30°$ C. for further use. The 9000 $\times$ $g$ supernatant was used immediately for benzopyrene hydroxylase activity. NADPH oxidase activity was determined spectrophotometrically (Cary Model 15) in a reaction mixture containing 0·1 ml. of the microsome fraction, 0·2 M phosphate buffer, pH 7·4, 100 $\mu$moles nicotinamide and

0·25 μmoles NADPH in a total volume of 3·0 ml. The volume of the reaction mixture was adjusted by the addition of the phosphate buffer and NADPH was omitted from the blank determination. The rate of decrease of adsorption peak at 340 nm. (Gillette *et al.*, 1957) was taken as a measurement of NADPH oxidase.

Microsomal NADPH-$Fe(CN)_6$ reductase was measured in a reaction mixture containing 0·1 ml. of the microsome fraction, 200 mμ moles potassium ferricyanide and 300 mμ moles of NADPH in a total volume of 3·0 ml. A phosphate buffer (0·05 M, pH 7·7) was used to obtain a final volume of 3 ml. Ferricyanide was not added to the blank. For this reaction, the decrease in absorbancy at 420 nm. was used to determine the activity (Williams and Kamin, 1962). NADPH cytochrome c reductase activity was also measured following the method of Williams and Kamin (1962). In the assays for NADPH oxidase, ferricyanide reductase and cytochrome c reductase, NADPH was added last to initiate the reaction.

Microsomal P-450 content was assayed by measuring the difference spectra of the preparation following the procedure of Omura and Sato (1964). Cytochrome $b_5$ was measured in a 3·0 ml. reaction mixture containing 800 mg. protein (105,000 × *g* fraction) and 2% NADH (10 lambda) (Fouts, 1969, personal communications). In all cases, the volume of the reaction mixture was kept constant by adjusting the amount of phosphate buffer added.

Benzopyrene hydroxylase was assayed fluorometrically by the method of Fouts (1969, personal communications). The Lowry procedure (Lowry *et al.*, 1951) was used to determine protein content of the microsomal fraction.

### RESULTS AND DISCUSSION

NADPH oxidase, NADPH-ferricyanide reductase and benzopyrene hydroxylase activities were greatly reduced in liver of breast-tumor-bearing animals as compared with controls (Table I). Our data for this breast tumor are in agreement with those of Kato and associates (1968) for another cancer. Cytochrome P-450 was markedly decreased, and to a lesser extent, cytochrome $b_5$ level was lower in tumor-bearing

TABLE I.—*Effect of Tumor on Enzymes of Microsomal Electron Transport and Benzopyrene Hydroxylating Systems in Wistar Rat Liver and in Breast Tumor Tissue*

| Enzyme | Liver (normal) | Liver (tumor bearing) | Change (%) | Liver (normal lactating) | Tumor |
|---|---|---|---|---|---|
| NADPH oxidase (mμmole/mg. protein/min.) | 11±1 | 6±1 | −45 | 9±0 | 2±0 |
| NADPH-ferricyanide reductase (mμmole/mg. protein/min.) | 24±1 | 14±1 | −42 | 21±2 | 2±0 |
| P-450 (mμmole/mg. protein) | 1·44±0·13 | 0·78±0·07 | −46 | 1·30±0·10 | 0 |
| Cytochrome $B_5$ ($\Delta$ $OD_{425-209}$/mg.) | 0·117±0·007 | 0·108±0·014 | −8 | 0·103±0·007 | 0 |
| Benzopyrene hydroxylase (mμmole hydrolyzed/mg. protein/hr) | 113±13 | 70±21 | −38 | 91±11 | 31±9 |

animals than in controls. Lactating animal liver was intermediate in value between that of the non-lactating and the tumor-bearing animal NADPH oxidase, NADPH ferricyanide reductase, and cytochromes P-450 and $b_5$.

The electron-transport oxidase system is absent from the tumor itself (Table I). NADPH oxidase, NADPH ferricyanide reductase, cytochromes P-450 and $b_5$, and benzopyrene hydroxylase were undetectable by the methods used. In the hydroxylase activity, as in other steps of the reaction sequence, the liver of lactating animals showed values intermediate between that of the non-lactating controls and the tumor-bearing animals.

Table II presents preliminary data referring to tissue distribution of the benzopyrene hydroxylase activity. A correspondence between hydroxylase activity and the known frequency of appearance of tumors in this experimental animal is in consonance with the data in Table II.

TABLE II.—*Tissue Distribution of Benzopyrene Hydroxylase in Normal, Lactating (normal) and Breast Tumor Bearing Wistar Rats*

| Animal | Tissue (9000 × g supernatant) | Activity (mμmole benzopyrene hydrolyzed/ mg. protein/hr |
|---|---|---|
| Normal | Liver | 113 ± 13 |
| | Kidney | 75 ± 35 |
| | Lung | 32 ± 7 |
| | Colon | 43 ± 13 |
| Lactating | Liver | 91 ± 11 |
| | Kidney | 70 ± 9 |
| | Lung | 31 ± 6 |
| | Colon | 39 ± 6 |
| Tumor bearing | Liver | 70 ± 21 |
| | Kidney | 55 ± 13 |
| | Lung | 42 ± 17 |
| | Colon | 37 ± 17 |

In the comparison of hepatoma-bearing animals with control Buffalo rats, under the conditions of these experiments, no significant difference existed when NADPH oxidase of normal liver was compared with tumor-bearing animal liver (Table III). In liver of 7777 tumor-bearing animals, however, NADPH-ferricyanide reductase and the CO-binding cytochrome (P-450) were both markedly

TABLE III.—*Effect of Morris Hepatoma (7777) on Enzyme of Microsomal Electron Transport and Benzopyrene Hydroxylating Systems in Buffalo Rat Liver and in Hepatoma Tissue*

| Enzyme | Liver (normal) | Liver (hepatoma bearing) | Change (%) | Hepatoma |
|---|---|---|---|---|
| NADPH oxidase (mμmole/mg. protein/min.) | 20 ± 9 | 21 ± 9 | +5 | 4·6 ± 0·3 |
| NADPH-cytochrome c reductase (mμmole/mg. protein/min.) | 198 ± 31 | 117 ± 25 | −10 | 4·8 ± 2·2 |
| NADPH-ferricyanide reductase (mμmole/mg. protein/min.) | 44 ± 9 | 23 ± 9 | −47 | 0·33 ± 0·12 |
| P-450 | 1·28 ± 0·42 | 0·85 ± 0·17 | −33 | — |
| Benzopyrene hydroxylase (mμmole hydrolyzed/mg. protein/hr) | 146 ± 26 | 124 ± 24 | −15 | 55 ± 25 |

reduced and NADPH-cytochrome c reductase was slightly reduced. P-450 content of the hepatoma-bearing animals showed a mean 33% lower than that of the mean of normal animals of the same strain. The NADPH-ferricyanide reductase was 47% less in the hepatoma-bearing animal than in normal Buffalo rat liver. The NADPH-cytochrome c reductase was also 10% less in the hepatoma-bearing rat liver. Electron transport enzymes obtained from the tumor tissue were essentially equal to zero.

Benzopyrene hydroxylase activity is lower in hepatoma-bearing liver compared to normal liver. Normal liver enzyme activity is 15% (mean) more than that of hepatoma-bearing liver. Hepatoma tissue itself has little benzopyrene hydrolyzing activity and other electron transport enzyme activities when compared to liver enzyme (Table III).

Our results, like those of Kato and associates, indicate that hepatoma 7777, like Walker carcinosarcoma 256, can be correlated with a lower level of liver microsomal hemoprotein and a decreased enzymatic activity of some components of the NADPH-linked electron transport system. The present study does not allow an ontogenetic interpretation of the lower activity of elements of the presumed detoxication system.

Presumably, microsomal oxidations serve in nature to lower the effect of environmental carcinogens upon the organism by oxidations which yield non-toxic products. The alternative interpretation of the lower levels of certain elements of the NADPH-electron transport system in tumor-bearing rats, as a casual relationship in the development of the tumor or secondary consequence, cannot at this point be discriminated.

The results of P-450 measurements, NADPH oxidase and ferricyanide reductase on liver microsomes as a function of radiation dose are shown in Fig. 1. It is apparent that an increase of 140% in P-450 content per milligram of protein occurs

FIG. 1.—Relationship between P-450 content and NADPH oxidase activity of liver microsomes and whole-body radiation exposure.

FIG. 2.—Relationship between $Fe(CN)_6 \equiv$ reductase activity of liver microsomes and whole-body radiation exposure.

as a function of doses up to the level of 1760 rad. The relationship between NADPH oxidase content of liver microsomes and the amount of radiation exposure indicated 100% increase in enzyme activity up to 1760 rad. total dose. However, there appears to be a more pronounced increase (90%) following lower dose level exposure and then only minor increases in NADPH oxidase content as the dose level is increased. Thus, by the measurement of these two parameters, one would be led to believe that radiation exposure should enhance the ability to metabolize materials by the mixed-function oxidase route if one considers that the two parameters measured are intermediates within this pathway. However, the ferricyanide reductase content of the microsomes as a function of radiation exposure has presented a different picture (Fig. 2). There is a remarkable decrease in enzyme activity upon exposure to 440 rad. dose. The activity tends to remain constant following exposure to higher rad. doses up to 1760 rad. which is the maximum dose in this experiment. Thus, while it would appear that radiation exposure leads to a slight, if irregular, increase in P-450 content and NADPH oxidase content of the microsomes, the ability of this system to transfer the electron to an artificial substrate (ferricyanide) is reduced by radiation exposure. These results have been verified with respect to the effect of whole-body irradiation on benzopyrene hydroxylation (Delwaide *et al.*, 1969). It would thus appear that the radiation-induced lesion in the adult rat represents a blocking of mixed-function oxidation at a point other than the presently recognized rate limiting steps.

It is also interesting to note that the tissue source (liver) was removed within 1 hour following the exposure to radiation. Thus, the observed changes represent short-term effects with respect to protein synthesis. The observation of such marked variation in a relatively short time following exposure tends to support the findings that this system is undergoing extremely rapid turnover with respect to protein (Gelboin, 1970, personal communications).

This research was supported by USPHS grants CA 08023 (Cancer Research Center) and CA 10729 (Harold P. Morris).

### REFERENCES

DeDuve, C., Wattiaux, R. and Baudhuin, P.—(1962) *Adv. Enzymol.*, **24**, 291.
Delwaide, P., Rondia, D. and Heusghem, C.—(1969) *Biochem. Pharmac.* **18**, 959.
Gillette, J. R., Brodie, B. B. and LaDu, B. N.—(1957) *J. Pharmac. exp. Ther.*, **119**, 532.
Kato, R., Frontino, G. and Vassanelli, P.—(1963) *Experientia*, **19**, 31.
Kato, R., Takanaka, A. and Takahashi, A.—(1968) *Gann*, **59**, 83.
Lowry, O. H., Rosenbrough, N. J., Farr, A. L. and Randall, R. J.—(1951) *J. biol. Chem.*, **193**, 265.
Miller, E.—(1970) Tenth International Congress, Historical Review and Perspectives, p. 2.
Omura, T. and Sato, R.—(1964) *J. biol. Chem.*, **239**, 2370.
Siekevitz, P.—(1963) *A. Rev. Physiol.*, **25**, 15.
Sugimura, T., Matsushima, T., Kawachi, T., Hirata, Y. and Kawabe, S.—(1966) *Gann*, **1**, 143.
Williams, C. H. and Kamin, H.—(1962) *J. biol. Chem.*, **237**, 587.